# Percutaneous coronary intervention for chronic total occlusion improved prognosis in patients with renal insufficiency at high risk of contrast-induced nephropathy

**DOI:** 10.1038/srep21426

**Published:** 2016-02-22

**Authors:** Yong Liu, Yuanhui Liu, Hualong Li, Yingling Zhou, Wei Guo, Chongyang Duan, Shiqun Chen, Pingyan Chen, Ning Tan, Jiyan Chen

**Affiliations:** 1 Department of Cardiology, Guangdong Cardiovascular Institute,Guangdong Provincial Key Laboratory of Coronary Disease, Guangdong General Hospital, Guangdong Academy of Medical Sciences, School of Medicine, South China University of Technology, Guangzhou, China; 2 National Clinical Research Centre for Kidney Disease, State Key Laboratory of Organ Failure Research, Department of Biostatistics, School of Public Health and Tropical Medicine, Southern Medical University, Guangzhou, China; 3 Department of Biostatistics, South China College of Cardiovascular Research, Guangdong Society of Interventional Cardiology, Guangzhou, China

## Abstract

We investigated whether attempted percutaneous coronary intervention (PCI) for chronic total occlusion (CTO) would improve the prognosis in patients with renal insufficiency at high risk of contrast-induced nephropathy (CIN). We analyzed 2,330 consecutive patients with renal insufficiency with or without CTOs who underwent coronary angiography or PCI from prospectively collected data. The long-term death and risk of CIN were evaluated among three groups: patients without CTOs (group A, n** = **1,829), patients with un-attempted PCI for CTOs (group B, n** = **142), and patients who underwent attempted PCI for CTOs (group C, n** = **359). Overall, group B and group C (successful rate, 89%) patients had similar renal function and were not significantly associated with an increased risk of CIN (adjusted odds ratio [OR]** = **0.88, 95% confidence interval [CI]: 0.41–1.93, *P*** = **0.758). During a 2.33-year period (median), multivariate analysis demonstrated that attempted PCI for CTOs was independently associated with lower mortality (adjusted hazard ratio for death: 0.38, 95% CI: 0.18–0.83; *P*** = **0.015). Attempted PCI for CTOs improved the long-term prognosis in patients with high-risk renal insufficiency and did not increase the risk of CIN.

With great advances in expertise and technologies, successful percutaneous coronary intervention (PCI) for chronic total occlusion (CTO) was shown to be associated with reduced long-term mortality in a recent study^1^. Guidelines recommend PCI for patients with mild and moderate chronic kidney disease (CKD), but not in patients with advanced CKD^2^. The impact of CKD on long-term outcomes seems to be greater than that of CTO in patients undergoing primary PCI^3^. Thus, it is unknown whether attempted PCI for CTO is beneficial for patients with renal insufficiency.

In patients with renal insufficiency undergoing PCI, the incidence of contrast-induced nephropathy (CIN), which is associated with prolonged hospitalization and an increased risk of death and healthcare costs, is as high as 30%^4^,^5^,^6^,^7^,^8^. Moreover, in patients with CTOs, even with a relatively good renal function, the rate of CIN (an underestimated definition in the 24 h) was also as high as 6.16%^9^. Few studies have investigated the risk of CIN in patients with both attempted PCI for CTOs and renal insufficiency, who need a high dose of contrast and may have more risk factors such as diabetes and advanced age.

Thus, we investigated whether attempted PCI for CTOs will improve prognosis in patients with renal insufficiency at high risk of CIN.

## Results

### Patients’ baseline clinical and procedural characteristics

**Figure 1:**
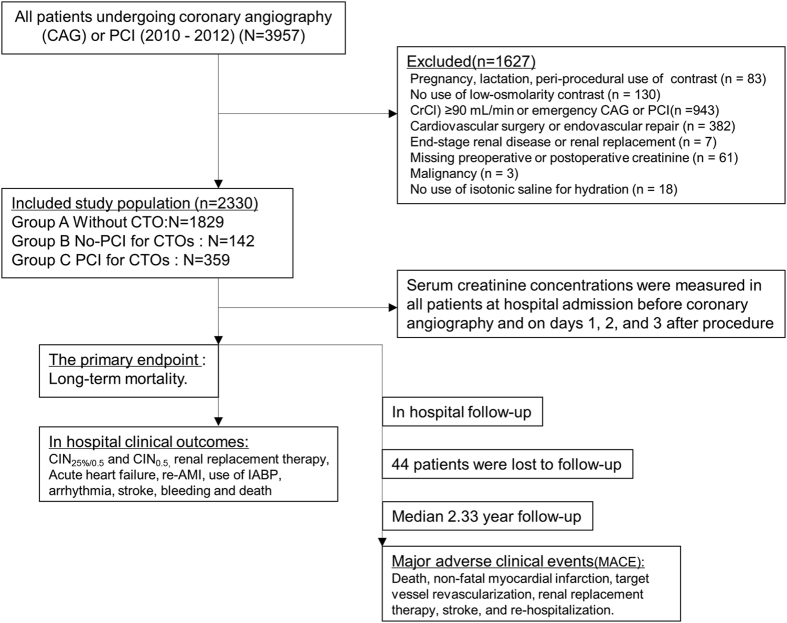
Flow Diagram of Patients with Chronic Kidney Disease Undergoing Coronary Angiography or PCI. A** = **without CTO; B** = **un-attempted CTO-PCI; C** = **attempted CTO-PCI; CI-AKI** = **contrast-induced acute kidney injury; CIN** = **contrast-induced nephropathy; CrCl** = **creatinine clearance; CTO** = **chronic total occlusion; IABP** = **intra-aortic balloon pump; PCI** = **percutaneous coronary intervention; re-AMI** = **relapse acute myocardial infarction.

Overall, 2,330 patients with renal insufficiency (73.6% men; age, 66 ± 10 years) were included in the final analysis (Fig. 1). There was a higher percentage of reduced left ventricular ejection fraction (LVEF) and (LVEF <40%) in patients with CTOs (55.61 ± 13.75 vs. 59.54 ± 12.49, *P* <.001; 13.8% vs. 9.7%, *P*** = **0.013). Patients who underwent attempted CTO-PCI (successful rate, 89%) had slightly better renal function than those that did not undergo attempted CTO-PCI, but it was not significantly different (CrCl: 62.33 ± 16.79 vs. 59.40 ± 16.63 mL/min, *P*** = **0.079). Furthermore, patients who underwent attempted PCI for CTOs showed a significantly lower rate of previous coronary artery bypass grafting (CABG: P <0.001) and higher usage of statins than those who did not undergo attempted PCI for CTOs (*P*** = **0.039) (Table 1). However, the mehran risk score, the incidence of previous myocardial infarction (MI) and diabetes mellitus, the usage of angiotensin-converting enzyme inhibitors/angiotensin receptor blocker (ACEI/ARB) were similar between patients who undergo attempted PCI for CTOs and those who did not undergo attempted PCI for CTOs (Table 1).

The angiographic and procedural characteristics are presented in Table 2. The presence of multi-vessel disease was significantly higher in patients with a CTO than in those without a CTO (88% vs. 58.5%, *P *< 0.001). There were a higher percentage of patients with a V/CrCl (ratio of contrast volume to creatinine clearance) of >2.62 who underwent attempted PCI for CTOs (49.9% vs. 31.7%, *P *< 0.001). Peri-procedural hydration volume was not significantly higher in group C than in group B (mean: 816 ± 452 vs. 809 ± 448mL, P = 0.891), and the same was found for the high hydration volume (<960mL, 64% vs. 62%, P = 0.625). In addition, in 96% of cases, an antegrade approach was followed, and in 4%, a retrograde approach was followed. However, the hydration volume was not significantly different among the three groups.

### In-hospital clinical events and predictors of CIN

Overall, CIN developed in 7.4% of patients (168/2,274). There was no difference in the prevalence of CIN among groups. For the different renal insufficiency categories, the prevalence of CIN was 5.8%, 8.5%, and 17.4% among the low-risk, moderate-risk, and high-risk categories, respectively. Furthermore, the incidence of renal replacement therapy, in-hospital mortality, and other adverse events were not higher in patients who underwent attempted PCI for CTOs than in the other groups (Table 3).

### Univariable analysis and multivariable analysis of CIN risk predictors

#### A comparison of patients who underwent attempted PCI for CTOs and those who did not undergo attempted PCI for CTOs (group C vs. B) for predicting CIN_25%/0.5_ and CIN_0.5_

**Figure 2:**
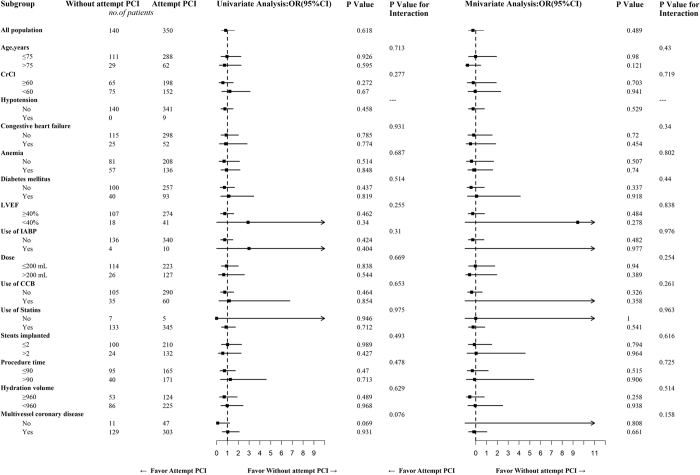
Selected Subgroup Analyses for Patients with Chronic Total Occlusion (CTO) (Attempted Percutaneous Coronary Intervention [PCI] vs. un-attempted PCI for CTO). CCB** = **calcium channel blockers; CrCl** = **creatinine clearance; Dose** = **contrast volume; IABP** = **intra-aortic balloon pump; LVEF** = **left ventricular ejection fraction.

Attempted PCI for CTOs was not significantly associated with an increased risk of CIN_25%/0.5_ compared to un-attempted PCI for CTOs (odds ratio [OR]** = **0.88, 95% CI: 0.41–1.93, *P*** = **0.758) (Table 4), which was similar to that found for the prespecified risk categories of patients (i.e., age, the hydration volume, CrCl, and congestive heart failure) (Fig. 2) and for the definition of CIN_0.5_ (Table 5).

### Selected Subgroup Analyses

Patients who underwent CTO-PCI were not significantly associated with an increased risk of CIN_25%/0.5_ compared to those who did not undergo CTO-PCI, and the prespecified risk categories of patients (i.e., age, hydration volume, CrCl, hypotension, congestive heart failure, anemia, diabetes mellitus, LVEF, IABP, contrast volume, calcium channel blocker, statin, the number of stents used, procedural duration, and multivessel coronary disease) (Fig. 2).

#### A comparison of patients with CTO and without CTO (group C vs. A and group B vs. A) for predicting CIN_25%/0.5_ and CIN_0.5_

Multivariable logistic regression analysis showed that attempted PCI for CTOs was not significantly associated with an increased risk of CIN_25%/0.5_ (group C vs. group A: OR** = **1.18; 95% CI: 0.72–1.93; *P*** = **0.513) (Table 4), which was similar to that found for the other definitions of CIN (CIN_0.5_) (Table 5). Multivariate analysis showed that female sex, age >75 years, a hydration volume <960 mL, and IABP were independent predictors of CIN_25%/0.5_ (Table 4).

Similarly, patients who did not undergo attempted PCI for CTOs (group B vs. group A: *P*** = **0.414) were not significantly associated with an increased risk of CIN_25%/0.5_ (Table 4), which was similar to that found for CIN_0.5_ (Table 5).

### Follow-up

**Figure 3:**
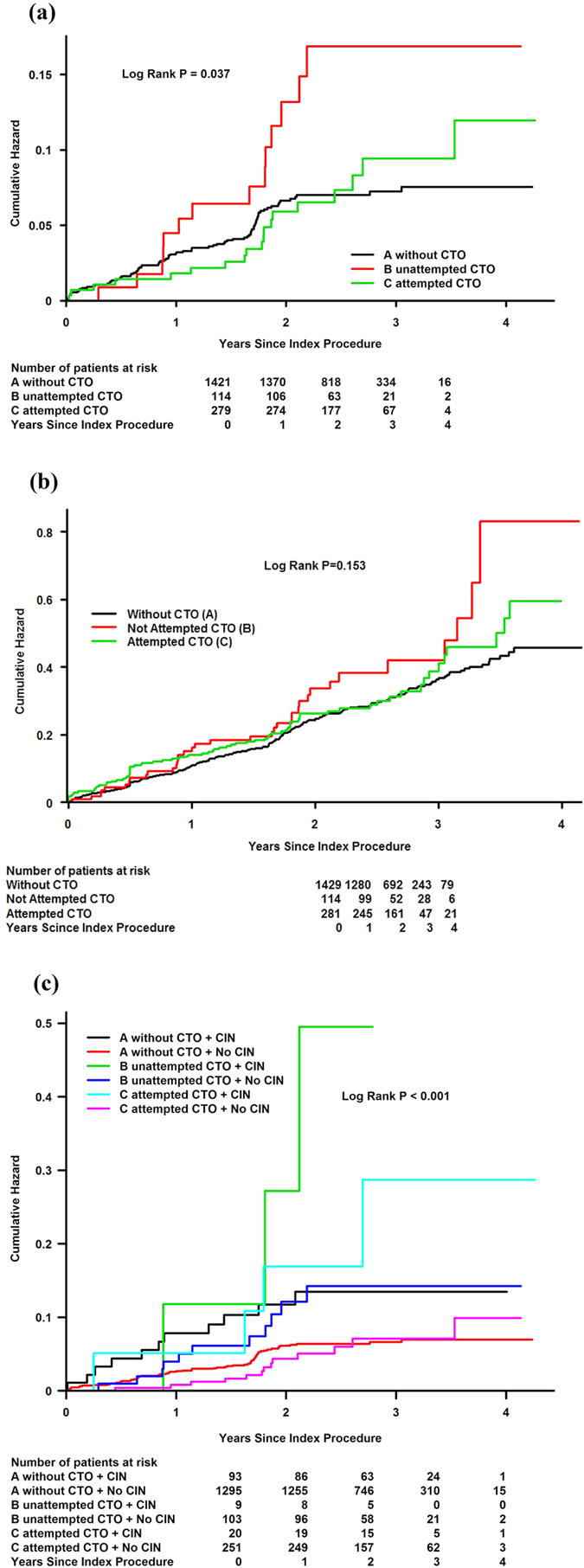
Kaplan-Meier Curves Showing Cumulative Probability of Mortality and Major Adverse Clinical Events. (**a**) Kaplan-Meier curves showing cumulative probability of mortality. (**b**) Kaplan-Meier curves showing cumulative probability of major adverse clinical events. (**c**) Kaplan-Meier curves showing cumulative probability of mortality.

In this cohort, the median follow-up was 2.33 years (interquartile range: 1.8–3.2 years). Kaplan-Meier curves for the clinical endpoints are shown in Fig. 3. Patients who underwent attempted PCI for CTOs had lower rates of mortality than those without attempted PCI for CTOs, but higher rates of mortality than those without CTOs (*P*** = **0.037). Particularly, patients with CIN who underwent attempted PCI for CTOs did not show a significantly higher risk of death than those without CIN (*P*** = **0.396).

#### A comparison of patients who underwent attempted PCI for CTOs and those who did not undergo attempted PCI for CTOs (group C vs. group B) for predicting mortality

**Figure 4:**
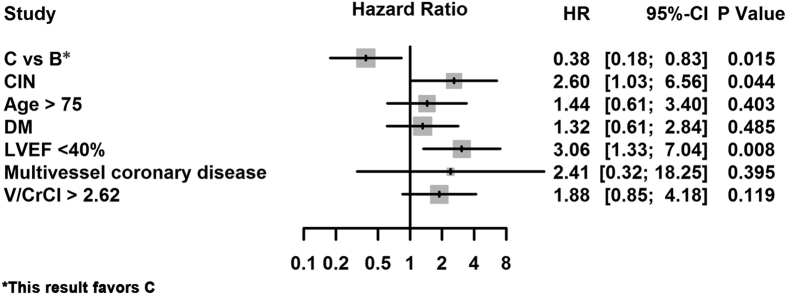
Adjusted Hazard Ratios of the Cox Analysis for Mortality. Group B** = **un-attempted CTO-PCI; Group C** = **attempted CTO-PCI; DM** = **diabetes mellitus; LVEF** = **left ventricular ejection fraction; V/CrCl** = **ratio of contrast volume to creatinine clearance.

After adjusting for CIN_25%/0.5_, multivariate Cox regression analysis showed that attempted PCI for CTOs (group C vs. group B) was significantly associated with a reduced risk of death (adjusted hazard ratio [HR]: 0.38, 95% CI: 0.18–0.83, *P*** = **0.015). The independent risk factors of death included CIN_25%/0.5_ and LVEF <40% (Fig. 4).

## Discussion

This was the first retrospective analysis, based on prospectively collected data,that evaluated the effect of attempted PCI for CTOs on the risk of CIN and the prognosis in high-risk patients with renal insufficiency. Our observational data showed that attempted PCI for CTOs had a high success rate (89%), and it still improved the long-term prognosis without increasing the risk of CIN in patients with renal insufficiency.

Patients who underwent attempted PCI for CTOs presented with slightly better renal function, but a higher contrast medium volume and higher percentage of V/CrCl of >2.62 than those that did not undergo attempted PCI for CTOs. Regardless of the contrast media insult during the procedure (PCI or no-PCI), the contrast volume of attempted PCI was more than un-attempted PCI for CTOs in this study. Additionally, a higher volume of contrast medium is associated with an increased risk of CIN due to hypoxia in the renal medulla and a direct toxic effect on renal cells^10^. Moreover, there significantly more complicated procedure, which includes more stents used, a longer total stent length, a longer procedural duration, results in higher volumes of contrast medium in patients who underwent attempted CTO-PCI than in those who did not undergo attempted PCI for CTO. Pre-existing renal dysfunction is also an independent risk factor of CIN^11^,^12^,^13^. We found that using a contrast dose based on the estimated renal function with a planned V/CrCl ratio of <3.7 (grams of iodine [g-I]/CrCl <1.37), preferably of <2.62 (g-I/CrCl <0.97), may be valuable for reducing the risks of CIN and even death after PCI^14^,^15^. However there was no significant difference in the Mehran score and risk factors, including IABP, anemia, and diabetes mellitus between patients who did or did not undergo attempted PCI for CTOs, which may mainly contribute to a similar risk of CIN^8^,^16^,^17^. In the present study, patients with CTO who did or did not undergo attempted PCI received a similar hydration volume, which may be the most effective measure to prevent CIN after PCI^6^,^18^. In addition, we could not evaluate the effect of individual oral hydration, which may be as effective as intravenous rehydration for preventing CIN^19^,^20^.

In Aguiar-Souto’s study (9), the incidence of CIN following PCI for CTO was as high as 6.16%, and Yu-Sheng *et al.*^21^. found a similar prevalence of CIN (5.4%) after CTO PCI. Both of these studies were consistent with our study, in which the occurrence of CIN was 8.6%. Our study had a relatively high percentage of CIN. Our patients had worse renal function, which significantly increased the risk of CIN^11^. Additionally, Aguiar-Souto’s study documented the creatinine levels as a biomarker of the study end point at baseline and 24 h after the procedure, when the peak level may be missed, causing the incidence of CIN to be underestimated^22^. Collected creatinine levels may be more reliable at 48 h and 72 h after the procedure in the present study.

Our study was consistent with a previous study that demonstrated that attempts to achieve recanalization of CTO in patients with CKD may not increase the risk of CIN; however, it lacked sufficient power to evaluate the long-term outcome due to a relatively small study population^23^. The long-term benefit of attempted PCI for CTOs in patients with CKD was similar to that in Mehran study^1^. Operator awareness, the application of specific algorithms, and the use of novel technology can contribute to minimizing the risk of contrast-related nephropathy^24^. In the present study, 76 patients in the group B underwent PCI for a non-CTO artery and not attempted PCI for CTO, but this may have also attributed to the good long-term prognosis. As all patients who did not undergo attempted PCI for CTOs did not receive complete revascularization, their mortality benefit was consistent across studies irrespective of the revascularization modality^25^. However, attempting PCI for CTOs did not increase the risk of CIN and reduce the improvement of the prognosis in these high-risk patients with renal insufficiency. Certainly, preventing CIN is still important during angiography, which includes PCI for CTOs^26^. With advancement in technology, it is recommended to perform attempted PCI for CTOs since it has a very high success rate in patients with CKD^27^. At our center, there are >10 well-experienced interventional cardiologists who perform PCI for CTOs with about a 90% success rate, thus they may experience less adverse events after this complex procedure^28^. In addition, attempted PCI for CTOs with a higher recanalization rate can improve patient’s long-term prognosis^29^,^30^.

The present study has several limitations. First, although the present study was prospective, it was observational in nature; thus, it cannot ascribe causality. Since it was also conducted at a single center among a relatively small patient population, it may not be representative of the wider population of patients who undergo PCI for CTOs. Second, measurements of the peak SCr levels may have been missed because of the variation in the measurement times, which were not standardized. This can potentially cause an underestimation of the true incidence of nephropathy in our study population. Third, there is not a standardized protocol for oral or intravenous periprocedural hydration. Finally, since data on oral hydration was missing, we were unable to exclude the effect of the whole hydration strategy on the risk of CIN in patients with renal insufficiency.

In conclusion, attempted PCI for CTOs still improved the long-term prognosis without increasing the risk of CIN in high-risk patients with renal insufficiency. When CIN risk stratification and not satisfaction, an individual contrast dose limit, and periprocedural hydration were also used to protect patient’s renal function, and attempted PCI for CTOs was considered in patients with renal insufficiency.

## Methods

### Subjects

In this prospective, observational, single-center study, we reviewed 3,957 consecutive patients who underwent coronary angiography or PCI between January 2010 and October 2012 according to the institutional protocol at our institution. As described in our previous study^31^, we included patients aged ≥18 years who agreed to stay in the hospital for 2–3 days after coronary angiography. In accordance with the updated European Society of Urogenital Radiology Contrast Media Safety Committee guidelines^32^, the exclusion criteria included pregnancy, lactation, intravascular administration of a contrast medium within the previous 7 days or 3 days postoperatively (n** = **83), no use of low-osmolarity contrast agents (n** = **130), creatinine clearance (CrCl) ≥90 mL/min or an emergency coronary angiography (n** = **943), cardiovascular surgery or endovascular repair (n** = **382), end-stage renal disease or renal replacement (n** = **7), missing preoperative or postoperative creatinine value (n** = **61), malignancy (n** = **3), and no use of isotonic saline for hydration (n** = **18).

Finally, 2,330 patients with renal insufficiency (CrCl* *< 90 mL/min) were retrospectively analyzed, of whom 501 were diagnosed with CTO. Among patients with CTO, 142 mainly underwent PCI of no occluded vessels, 359 underwent PCI for CTO. CTO was defined as thrombolysis in myocardial infraction grade 0 for >3 months with the presence of typical angina or reversible myocardial ischemia on a stress study^9^.

Follow-up events were carefully monitored and were recorded by trained nurses through office visits and telephone interviews at 1, 6, 12, and 24 months after coronary angiography. The mean follow-up time was 2.45 ± 0.82 years (median, 2.33 years; interquartile range, 1.78–3.19 years). All experiments were performed in accordance with the approved protocols, guidelines, and regulations. This study was approved by the Guangdong General Hospital institutional ethics research committee, and all patients provided written informed consent.

### Coronary angiography

Coronary angiography was performed according to general clinical practice using general guide catheters, guidewires, balloon catheters, and stents through the femoral or radial approach. The contrast dose was left to the discretion of the interventional cardiologist. All patients received 370 mg I/mL of nonionic, low-osmolality contrast agents, either lopamiron or ultravist. Subjects were treated according to the American Heart Association/American College of Cardiology Foundation guidelines^2^. According to the local institutional protocol^31^, serum creatinine concentrations were measured in all patients at hospital admission before coronary angiography and on days 1, 2, and 3 after coronary angiography.

The success criteria of PCI for CTOs was defined as a final diameter stenosis <30% with a TIMI flow grade 3 of the CTO vessel without death, or Q-wave myocardial infarction, or emergency coronary surgery. A Q-wave myocardial infarction was defined as new Q waves in two or more contiguous leads in addition to creatine kinase-myocardial band elevation. Creatine kinase-myocardial band fraction was routinely assessed 12 h after PCI in all patients or at least three times every 6h in patients with recurrent chest pain^33^.

The CrCl was calculated by applying the Cockcroft-Gault formula to the serum creatinine concentration^34^. Patients received a continuous intravenous infusion of isotonic saline at a rate of 1 mL/kg/h (or 0.5 mL/kg/h in cases of left ventricular ejection fraction of <40% or severe congestive heart failure) for at least 2–12 h before and 6–24 h after the procedure. IABP insertion should be considered in patients with hemodynamic instability/cardiogenic shock for peri-procedure support according to the recent guidelines^2^.

### Study endpoints

The primary endpoint was long-term mortality; the second endpoint was long-term major adverse clinical events (e.g., death, non-fatal myocardial infarction, target vessel revascularization, CIN requiring renal replacement therapy, stroke, and re-hospitalization). We also observed patients’ in-hospital clinical outcomes, including renal replacement therapy, acute heart failure, re-AMI, the use of an intra-aortic balloon pump (IABP), arrhythmia, stroke, bleeding and death. CIN_25%/0.5_ was defined as an increase in the serum creatinine level of >0.5 mg/dL (≥44.2 μmol/L) or of ≥25% from the baseline within 48–72 h of contrast exposure^35^. CIN_0.5_ was defined as an increase in the serum creatinine level of >0.5 mg/dL (≥44.2 μmol/L) from baseline within 48–72 h of contrast exposure.

### Statistical analysis

First, all the sample patients were divided into two groups: patients without CTOs and patients with CTOs. Then patients with CTOs were further divided into patients who did not undergo attempted PCI for CTOs and those who underwent attempted PCI for CTOs. Continuous variables are presented as mean ± standard deviation or median with interquartile range if they fit a normal or non-normal distribution, respectively. Comparisons between two groups were performed using Student’s t-test or the Wilcoxon rank-sum test. Pearson’s chi-square or Fisher’s exact tests were used, as appropriate, for categorical data, and the data are expressed as percentages. The incidence of CIN was compared among three groups using unadjusted and adjusted logistic regression analyses. Multivariable logistic regression analyses were also performed. Univariable analyses of mortality were performed using the log-rank test, and multivariable Cox regression analyses were performed. The data were analyzed on an available case basis, and missing data were not imputed. A two-tailed *P* value <0.05 was considered statistically significant. All data analyses were performed using SAS, version 9.4 (SAS Institute).

## Additional Information

**How to cite this article**: Liu, Y. *et al.* Percutaneous coronary intervention for chronic total occlusion improved prognosis in patients with renal insufficiency at high risk of contrast-induced nephropathy. *Sci. Rep.* **6**, 21426; doi: 10.1038/srep21426 (2016).

## Figures and Tables

**Table 1:** Baseline demographics and clinical features in patients with renal insufficiency.

Characteristic	Group A (n = 1,829)	Group B (n = 142)	Group C (n = 359)	P*-value	Pâ-value
Demographic					
Age, years	65.88 ± 9.63	66.30 ± 9.36	65.81 ± 9.65	0.604	0.881
Male sex, n (%)	1,303 (71.3%)	123 (86.6%)	287 (79.9%)	0.081	<0.001
Smokers, n (%)	613 (33.5%)	55 (38.7%)	141 (39.3%)	0.911	0.020
Hypertension, n (%)	1,106 (60.5%)	85 (59.9%)	226 (63.0%)	0.520	0.514
Diabetes mellitus, n (%)	414 (22.7%)	40 (28.2%)	95 (26.5%)	0.698	0.045
Hyperlipidaemia, n (%)	241 (13.2%)	15 (10.6%)	44 (12.3%)	0.596	0.407
Previous MI, n (%)	164 (9.0%)	32 (22.5%)	64 (17.8%)	0.228	<0.001
Previous CABG, n (%)	5 (0.3%)	13 (9.2%)	2 (0.6%)	<0.001	<0.001
Weight, kg	62.62 ± 9.61	64.11 ± 9.35	63.34 ± 9.74	0.423	0.053
Biochemical					
Baseline SCr, μmol/L	96.17 ± 42.84	101.92 ± 33.19	94.57 ± 27.13	0.020	0.756
Baseline CrCl, mL/min	61.49 ± 17.17	59.40 ± 16.63	62.33 ± 16.79	0.079	0.998
CKD stage, CrCl, mL/min				0.168	0.069
II (60–90), n (%)	1,016 (56.7%)	67 (47.2%)	199 (56.4%)		
III (30–60), n (%)	696 (38.8%)	71 (50.0%)	144 (40.8%)		
IV–V (<30), n (%)	81 (4.5%)	4 (2.8%)	10 (2.8%)		
Total cholesterol, mmol/L	4.31 ± 1.53	4.24 ± 1.25	4.32 ± 1.24	0.731	0.586
Anaemia, n (%)	624 (34.9%)	58 (41.4%)	136 (38.9%)	0.599	0.056
LDL-C, mmol/L	2.52 ± 0.86	2.62 ± 0.97	2.58 ± 0.88	0.718	0.208
Clinical Presentation					
Congestive heart failure, n (%)	255 (14.3%)	26 (18.3%)	54 (15.2%)	0.389	0.312
Hypotension, n (%)	23 (1.3%)	0 (0.0%)	11 (3.1%)	0.035	0.122
LVEF, (%)	59.54 ± 12.49	53.68 ± 14.02	56.37 ± 13.58	0.061	<0.001
LVEF <40%, n (%)	151 (9.7%)	19 (15.0%)	43 (13.3%)	0.648	0.013
Mehran risk score				0.623	0.028
≤5	1,260 (72.3%)	88 (62.9%)	230 (66.3%)		
6–10	351 (20.1%)	41 (29.3%)	84 (24.2%)		
11–15	88 (5.0%)	8 (5.7%)	21 (6.1%)		
≥16	44 (2.5%)	3 (2.1%)	12 (3.5%)		
Medication Therapy					
ACEI/ARB, n (%)	1,576 (86.2%)	125 (88.0%)	333 (92.8%)	0.089	0.020
ß-blockers, n (%)	1540(84.2%)	125(88.0%)	314(87.5%)	0.863	0.061
Calcium channel blockers, n (%)	356 (19.5%)	35 (24.6%)	61 (17.0%)	0.050	0.859
Statins, n (%)	1710(93.5%)	135(95.1%)	353(98.3%)	0.039	<0.001

Group A** = **without chronic total occlusion (CTO); Group B** = **un-attempted CTO-PCI; Group C** = **attempted CTO-PCI; MI** = **myocardial infarction; CABG** = **coronary artery bypass grafting; LVEF** = **left ventricular ejection fraction; SCr** = **serum creatinine; CrCl** = **creatinine clearance; CKD** = **chronic kidney disease; LDL-C** = **low-density lipoprotein cholesterol; ACEI/ARB** = **angiotensin-converting enzyme inhibitors/angiotensin receptor blocker;

P*, group B vs. group C;

P^â^, group A vs. group B + group C.

**Table 2:** Angiographic and procedural characteristics in patients with renal insufficiency.

Characteristic	Group A (n = 1,829)	Group B (n = 142)	Group C (n = 359)	P*-value	Pâ-value
Multivessel coronary disease, n (%)	1,070 (58.5%)	131 (92.3%)	310 (86.4%)	0.067	<0.001
Multiple CTO, ≥2		31 (21.8%)	69 (19.2%)	0.510	
CTO vessel					
RCA		63 (44.4%)	186 (51.8%)	0.133	
LAD		59 (41.5%)	164 (45.7%)	0.402	
LCX		54 (38.0%)	80 (22.3%)	<0.001	
LM		2 (1.4%)	3 (0.8%)	0.561	
No. of stents used, n	1.52 ± 1.24	1.35 ± 1.35	2.25 ± 1.29	<0.001	<0.001
Total stent length, mm	36.57 ± 32.30	32.88 ± 34.48	58.94 ± 36.97	<0.001	<0.001
Procedural duration, min	63.09 ± 41.55	74.64 ± 45.49	103.25 ± 51.24	<0.001	<0.001
Contrast volume, mL	119.54 ± 67.42	126.76 ± 71.84	169.29 ± 66.39	<0.001	<0.001
<100	645 (35.4%)	47 (33.1%)	22 (6.1%)		
100–200	905 (49.7%)	69 (48.6%)	208 (57.9%)		
>200	271 (14.9%)	26(18.3%)	129 (35.9%)		
Hydration volume, mL	776.17 ± 431.26	809.96 ± 447.75	816.08 ± 451.64	0.891	0.084
<960 mL, n (%)	1,219 (68.1%)	87 (61.7%)	228 (64.0%)	0.625	0.049
V/CrCl > 2.62, n (%)	521 (29.1%)	45 (31.7%)	176 (49.9%)	<0.001	<0.001

Group A = without chronic total occlusion (CTO); Group B = un-attempted CTO-PCI; Group C = attempted CTO-PCI; PCI = percutaneous coronary intervention; CKD = chronic kidney disease; CTO = chronic total occlusion; RCA = right coronary artery; LAD = left anterior descending; LCX = left circumflex coronary artery; LM** = **left main coronary artery;

V/CrCl = ratio of contrast volume to creatinine clearance; P*, group B vs, group C;

Pâ, group A vs. group B + group C.

**Table 3:** In-hospital clinical outcomes in patients with renal insufficiency.

Characteristic	Group A (n = 1,829)	Group B (n = 142)	Group C (n = 359)	P*-value	Pâ-value
CIN_25%/0.5_ (increase ≥25% or 0.5 mg/dL within 72 h), n (%)	124 (7.0%)	14 (10.0%)	30 (8.6%)	0.617	0.128
CKD stage II	54 (5.4%)	7 (10.8%)	13 (6.6%)	0.267	0.166
CKD stage III	55 (8.0%)	7 (9.9%)	15 (10.6%)	0.874	0.284
CKD stage IV–V	15 (18.8%)	0 (0.0%)	2 (20.0%)	0.334	0.689
CIN_0.5_ (increase 0.5 mg/dL within 72 h), n (%)	38 (2.1%)	5 (3.6%)	11 (3.1%)	0.809	0.144
CI-AKI (increase ≥50% or 0.3 mg/dL within 48 h), n (%)	124 (7.0%)	0 (0.0%)	2 (20.0%)	0.334	0.128
Renal replacement therapy, n (%)	11 (0.6%)	0 (0.0%)	2 (0.6%)	0.373	0.589
Acute heart failure, n (%)	26 (1.4%)	4 (2.8%)	6 (1.7%)	0.409	0.357
Re AMI, n (%)	6 (0.3%)	1 (0.7%)	0 (0.0%)	0.111	0.641
IABP, n (%)	30 (1.6%)	4 (2.8%)	13 (3.6%)	0.654	0.654
Arrhythmia, n (%)	33 (1.8%)	2 (1.4%)	8 (2.2%)	0.554	0.780
Stroke, n (%)	2 (0.1%)	2 (1.4%)	1 (0.3%)	0.140	0.036
Bleeding, n (%)	8 (0.4%)	2 (1.4%)	1 (0.3%)	0.140	0.641
Death, n (%)	15 (0.8%)	3 (2.1%)	2 (0.6%)	0.114	0.702

Group A = without chronic total occlusion (CTO); Group B = un-attempted CTO-PCI; Group C = attempted CTO-PCI; CIN = contrast-induced nephropathy; CKD** = **chronic kidney disease; CI-AKI** = **contrast-induced acute kidney injury; Re AMI** = **relapse acute myocardial infarction; IABP** = **intra-aortic balloon pump;

P*, group B vs. group C;

P^â^, group A vs. group B + group C.

**Table 4:** Multivariate analysis of contrast-induced nephropathy_25%/0.5_ risk predictors.

Variables	Univariable analysis			Multivariable analysis		
	OR	95% CI	P value	OR	95% CI	P value
B vs. A	1.49	0.83–2.66	0.181	1.33	0.67–2.65	0.414
C vs. A	1.26	0.83–1.90	0.285	1.18	0.72–1.93	0.513
C vs. B	0.84	0.43~1.64	0.618	0.88	0.41~1.93	0.758
Female sex	1.25	0.88–1.76	0.210	1.52	1.01–2.28	0.045
Age >75 years	2.16	1.52–3.08	<0.001	1.81	1.14–2.86	0.012
Hydration volume <960 mL	0.56	0.41–0.78	<0.001	0.66	0.44–0.98	0.040
ACEI/ARB	1.06	0.64–1.73	0.828	0.95	0.52–1.72	0.861
Anaemia	1.66	1.21–2.29	0.002	1.28	0.87–1.89	0.209
CHF	2.41	1.68–3.46	<0.001	1.53	0.98–2.41	0.064
CrCl	0.98	0.97–0.99	<0.001	1.01	0.99–1.02	0.287
DM	1.24	0.87–1.76	0.236	1.04	0.68–1.58	0.857
LVEF <40%	1.57	0.98–2.50	0.060	1.44	0.84–2.47	0.181
Contrast volume	1.00	1.00–1.00	0.838	1.00	1.00–1.00	0.849
Multivessel coronary disease	1.69	1.18–2.43	0.005	1.65	0.98–2.78	0.059
IABP	6.01	3.06–11.79	<0.001	3.25	1.47–7.20	0.004
Hypotension	5.06	2.21–11.61	<0.001	1.60	0.48–5.33	0.443
No. of stents used	1.06	0.93–1.20	0.366	0.96	0.80–1.15	0.684
Procedural duration	1.22	0.85–1.74	0.281	1.03	0.65–1.62	0.897
CCB	1.14	0.78–1.68	0.494	1.00	0.64–1.57	1.000

Group A** = **without chronic total occlusion (CTO); Group B** = **un-attempted CTO-PCI; Group C** = **attempted CTO-PCI; ACEI/ARB** = **angiotensin-converting enzyme inhibitors/angiotensin receptor blocker; CHF** = **congestive heart failure; CrCl** = **creatinine clearance rate; DM** = **diabetes mellitus; LVEF** = **left ventricular ejection fraction; IABP** = **intra-aortic balloon pump; CCB** = **calcium channel blockers.

**Table 5:** Multivariate analysis of contrast-induced nephropathy_0.5_ risk predictors.

Variables	Univariable analysis			Multivariable analysis		
	OR	95% CI	P value	OR	95% CI	P value
B vs. A	1.70	0.66–4.39	0.272	1.80	0.57–5.75	0.319
C vs. A	1.49	0.75–2.95	0.250	1.49	0.63–3.54	0.369
C vs. B	0.88	0.30~1.57	0.810	0.83	0.22~3.04	0.773
Females	1.08	0.59–1.98	0.797	1.25	0.61–2.56	0.541
Age >75 years	3.07	1.75–5.39	<0.001	1.10	0.53–2.29	0.789
Hydration volume <960 mL	0.30	0.17–0.52	<0.001	0.60	0.29–1.24	0.168
ACEI/ARB	0.66	0.32–1.37	0.269	0.84	0.32–2.20	0.727
Anaemia	3.19	1.80–5.64	<0.001	1.88	0.93–3.83	0.080
CHF	4.25	2.44–7.41	<0.001	2.07	1.01–4.22	0.046
CrCl	0.94	0.92–0.95	<0.001	0.95	0.93–0.98	<0.001
DM	1.13	0.61–2.10	0.689	0.71	0.33–1.54	0.388
LVEF <40%	1.73	0.80–3.75	0.165	0.93	0.35–2.47	0.879
Contrast volume	1.00	1.00–1.01	0.209	1.00	0.99–1.01	0.891
Multivessel coronary disease	1.68	0.90–3.16	0.106	0.66	0.26–1.69	0.388
IABP	11.17	4.90–25.46	<0.001	3.07	1.05–9.01	0.041
Hypotension	9.95	3.63–27.22	<0.001	1.94	0.36–10.38	0.440
No. of stents used	1.18	0.96–1.44	0.114	1.11	0.83–1.51	0.480
Procedural duration	2.03	1.16–3.57	0.013	1.23	0.57–2.68	0.595
CCB	1.37	0.73–2.60	0.329	1.10	0.51–2.37	0.800

Group A** = **without chronic total occlusion (CTO); Group B** = **un-attempted CTO-PCI; Group C** = **attempted CTO-PCI; ACEI/ARB** = **angiotensin-converting enzyme inhibitors/angiotensin receptor blocker; CHF** = **congestive heart failure; CrCl** = **creatinine clearance rate; DM** = **diabetes mellitus; LVEF** = **left ventricular ejection fraction; IABP** = **intra-aortic balloon pump; CCB** = **calcium channel blockers.
